# Supplementary material for: Facile One-Pot Multicomponent Synthesis of Pyrazolo-Thiazole Substituted Pyridines with Potential Anti-Proliferative Activity: Synthesis, In Vitro and In Silico Studies
Source: Molecules. 2021 May 22;26(11):3103. doi: 10.3390/molecules26113103 (PMC8196987; doi:10.3390/molecules26113103)
Supplement: Supplementary file 1 [file molecules-26-03103-s001.zip › molecules-1204761-supplementary.pdf]

Supplementary Data

Facile one-pot multicomponent synthesis of pyrazolo-thiazole substituted pyridines with potential anti-proliferative activity: Synthesis, *in vitro* and *in silico* studies

Islam H. El Azab[1*], Rania B. Bakr[2], and Nadia A.A. Elkanzi[3,4]

[1] Food Science and Nutrition Department, College of Science, Taif University, P.O. box 11099, Taif 21944, Saudi Arabia.

[2]Department of Pharmaceutical Organic Chemistry, Faculty of Pharmacy, Beni-Suef University, Beni-Suef, Egypt

[3] Chemistry Department, College of Science, Jouf University, P.O. Box 2014, Sakaka, Saudi Arabia.

[4]Chemistry Department, Faculty of Science, Aswan University, Aswan, P.O. box 81528, Aswan, Egypt.

*Correspondence: i.helmy@tu.edu.sa (I.H.E.)

Received: date; Accepted: date; Published: date

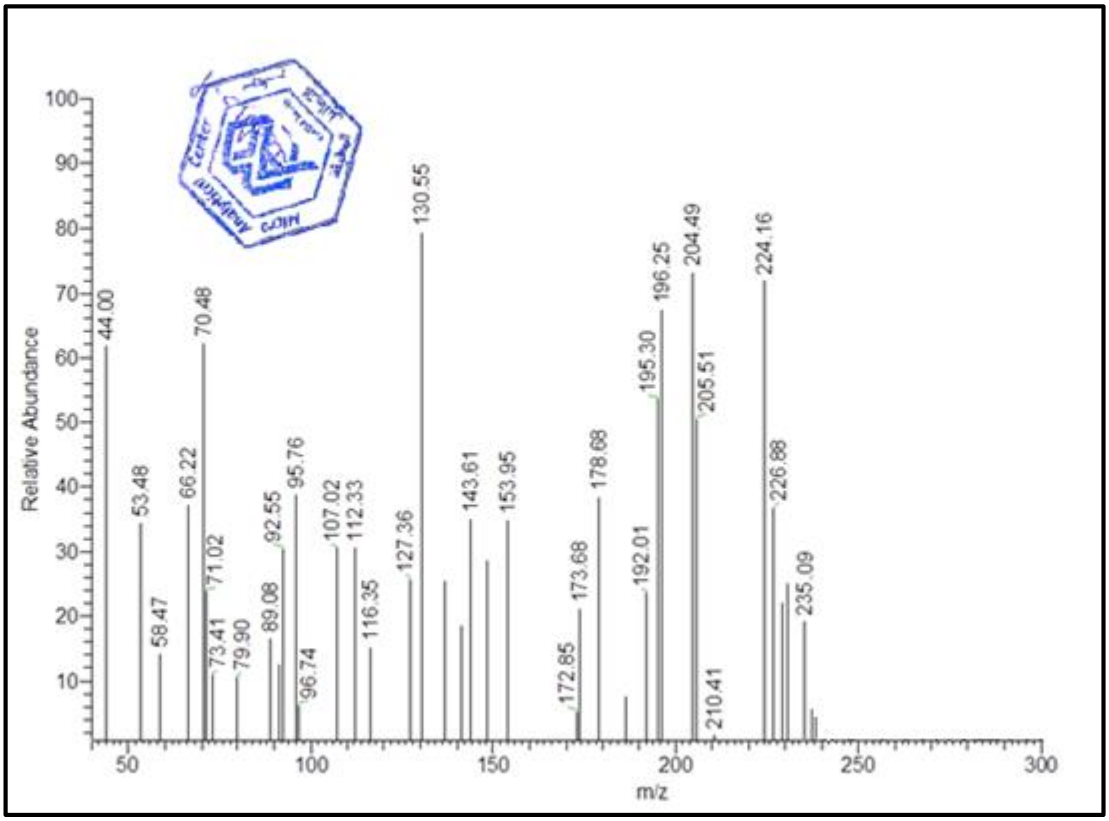

Mass of compound 2

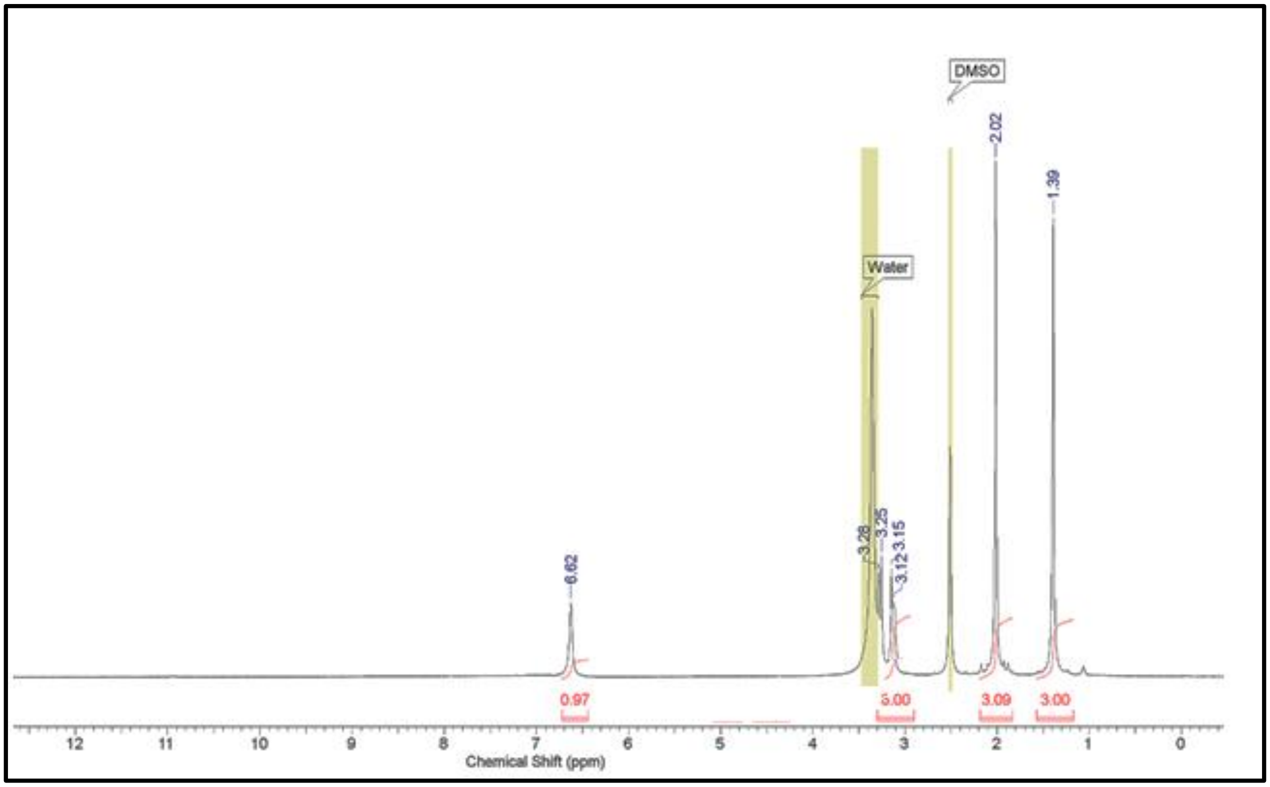

^{1}H NMR of compound 2

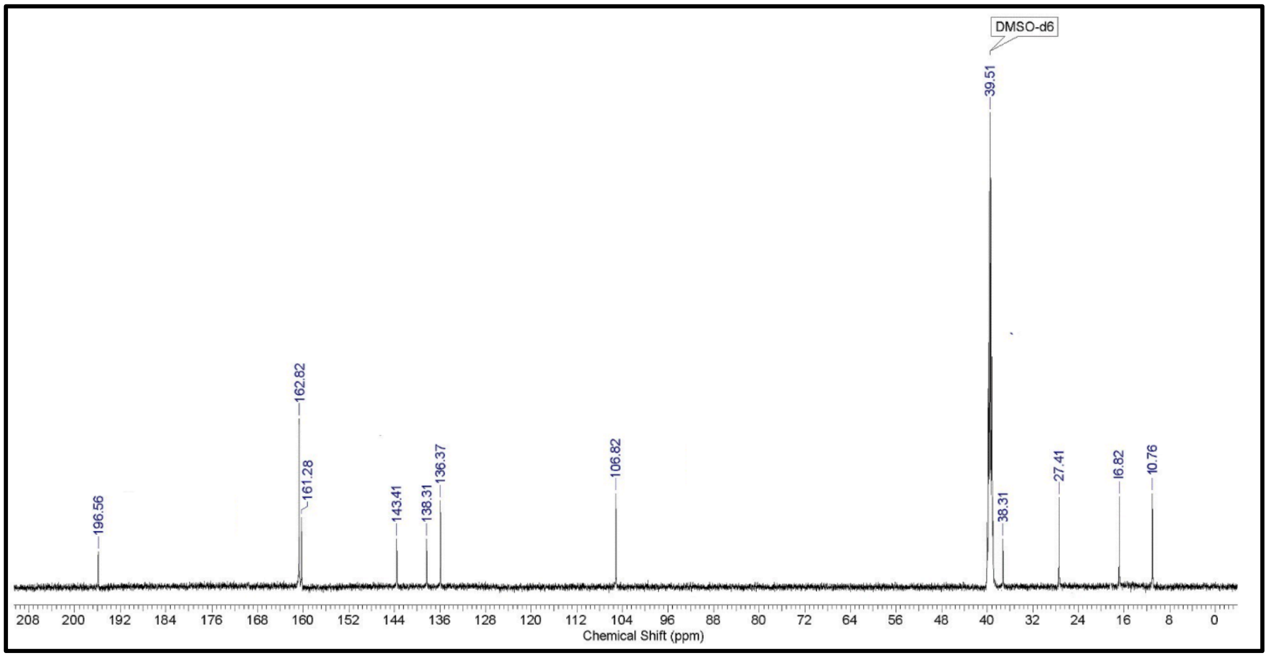

^{13}C NMR of compound 2

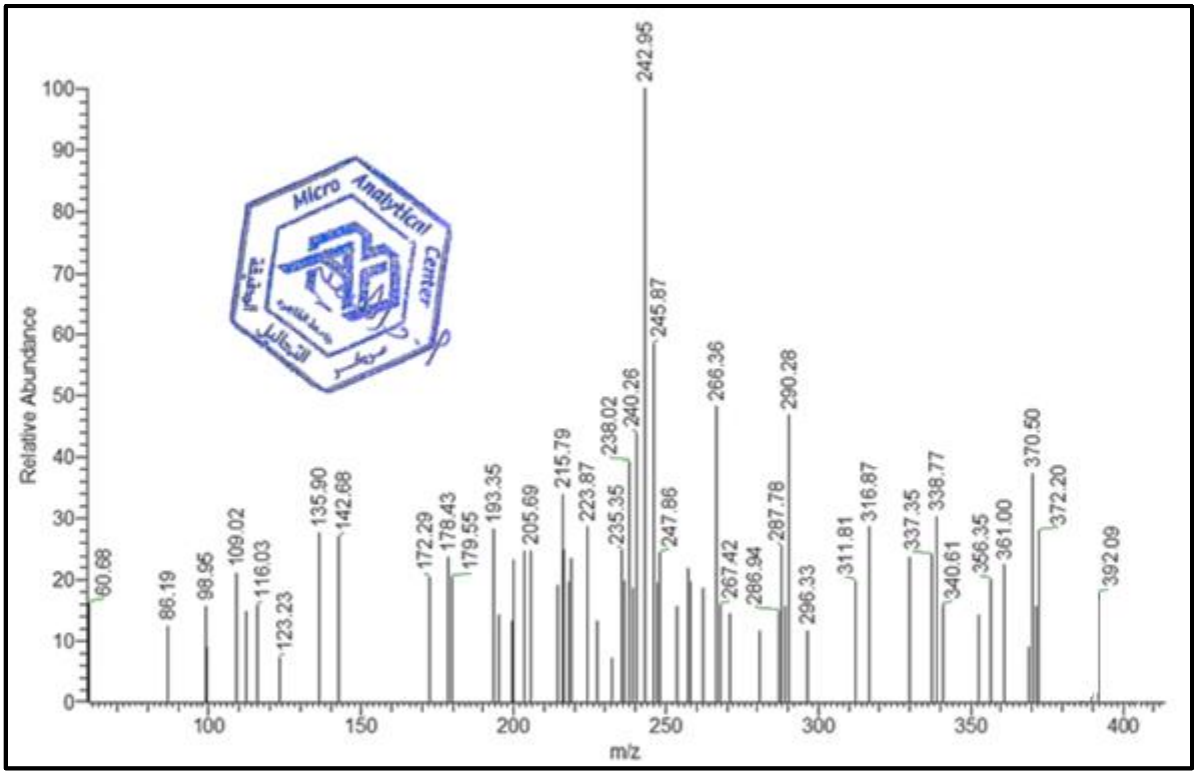

Mass of compound 4

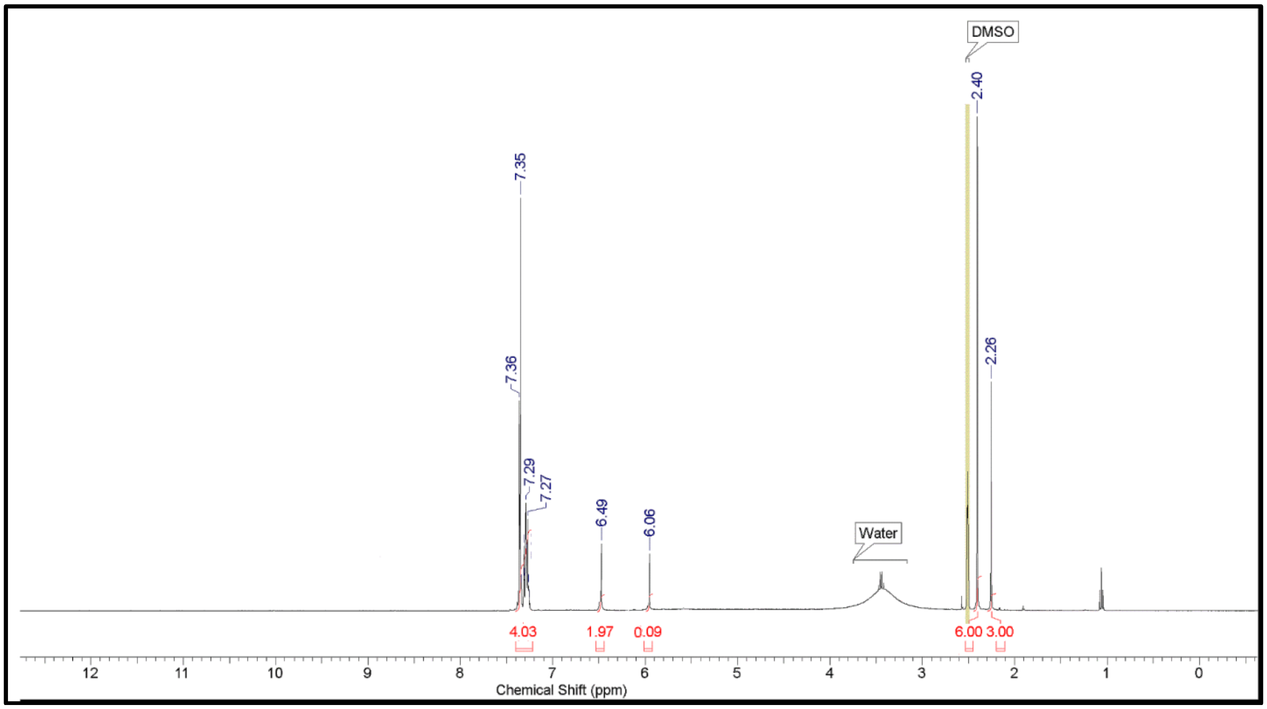

^{1}H NMR of compound 4

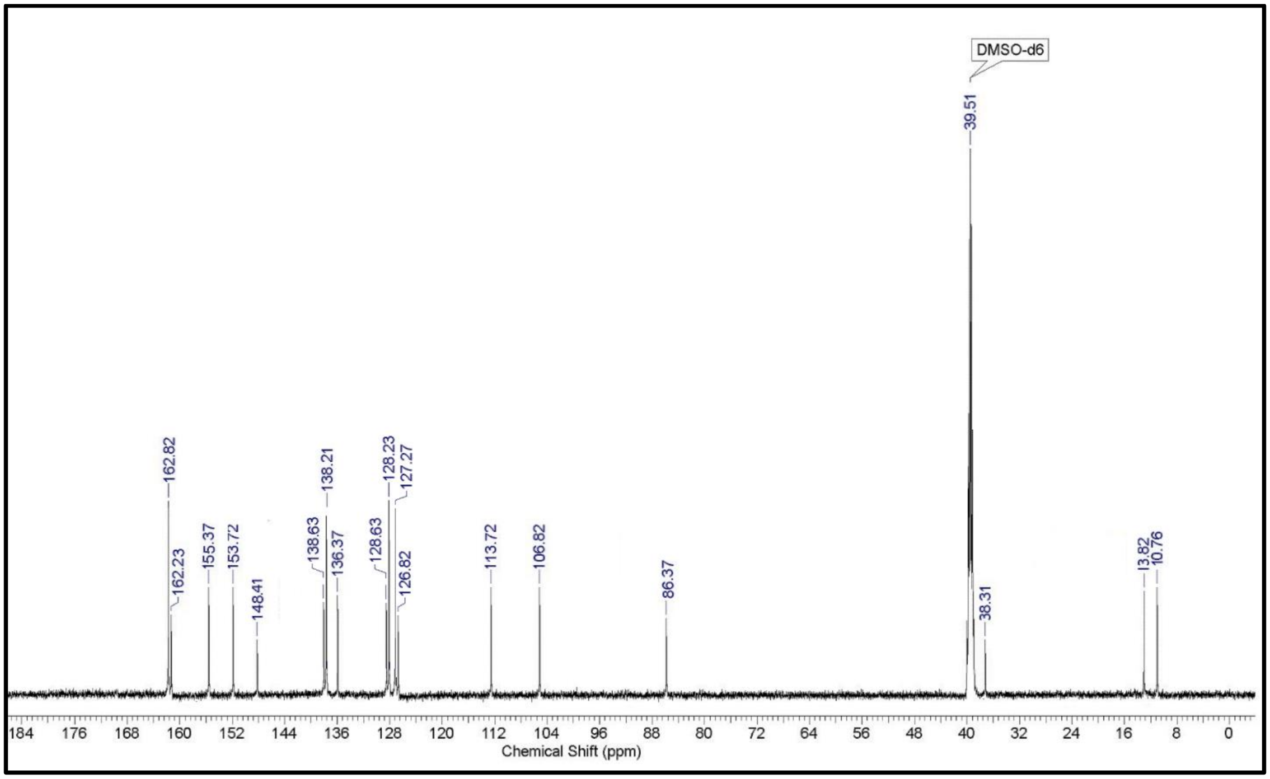

^{13}C NMR of compound 4

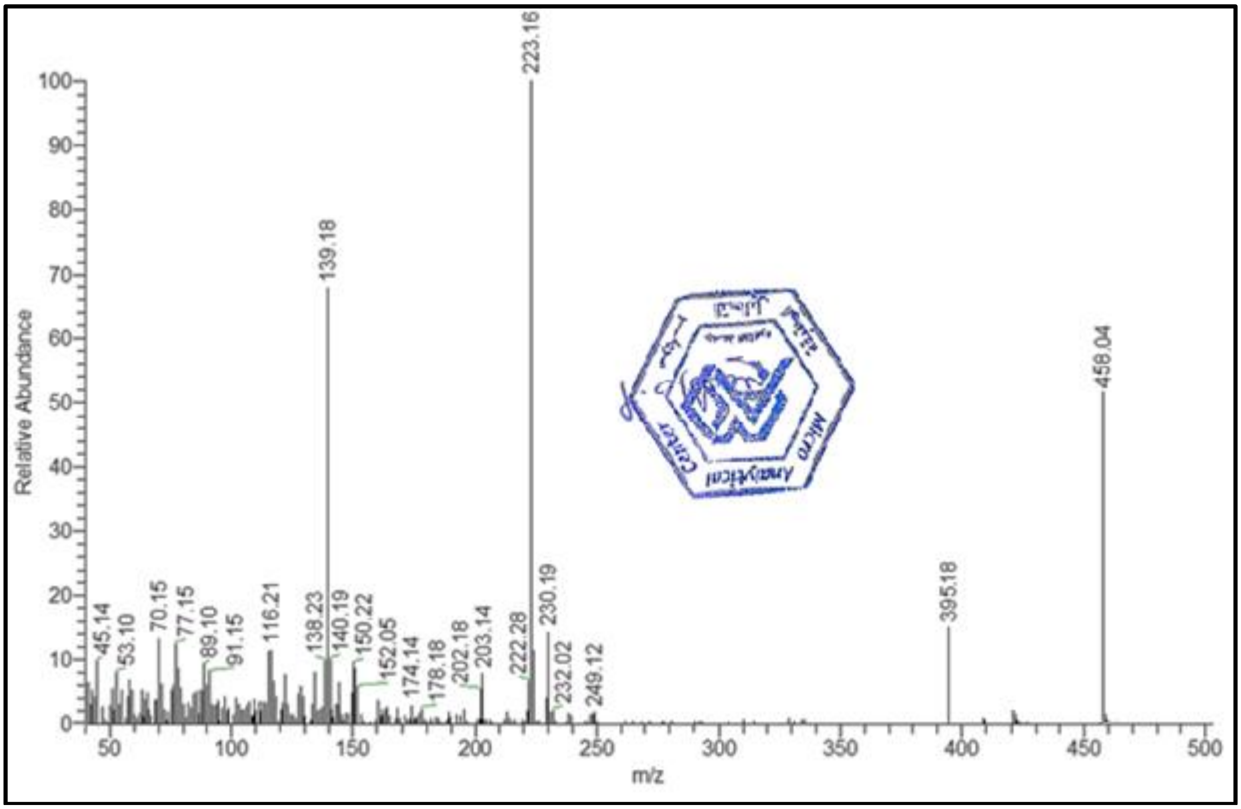

Mass of compound 6

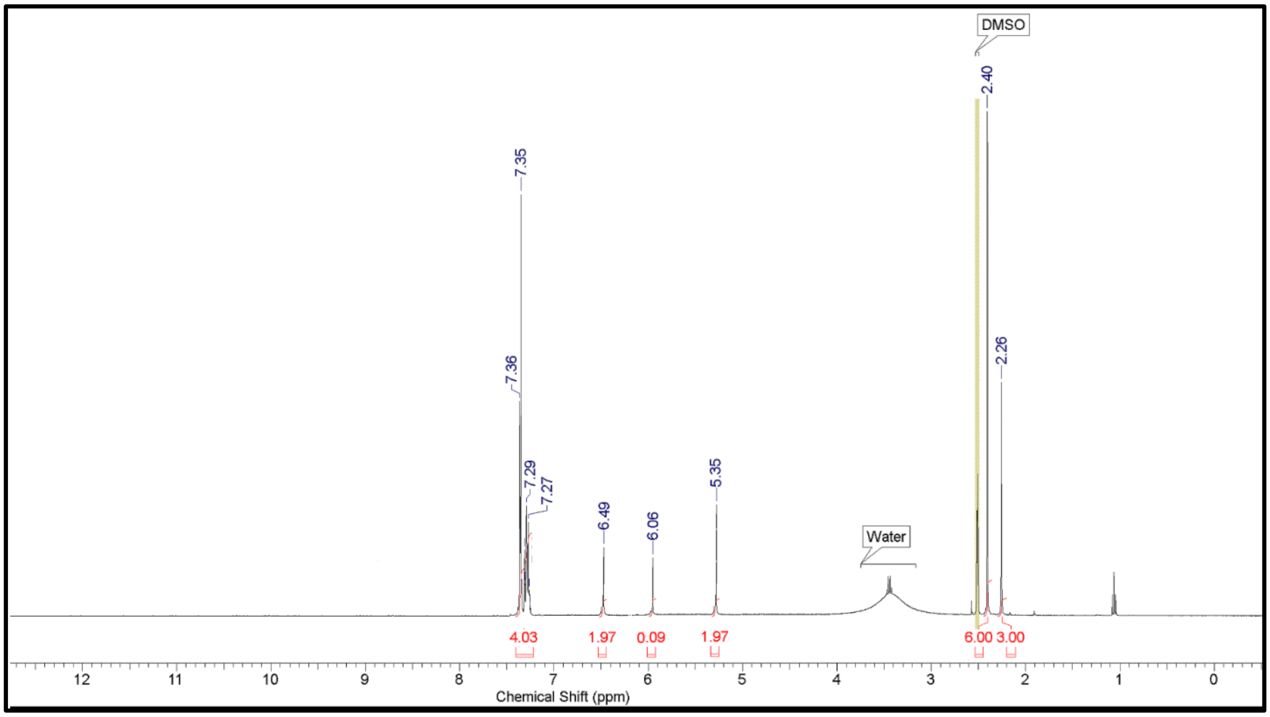

^{1}H NMR of compound 6

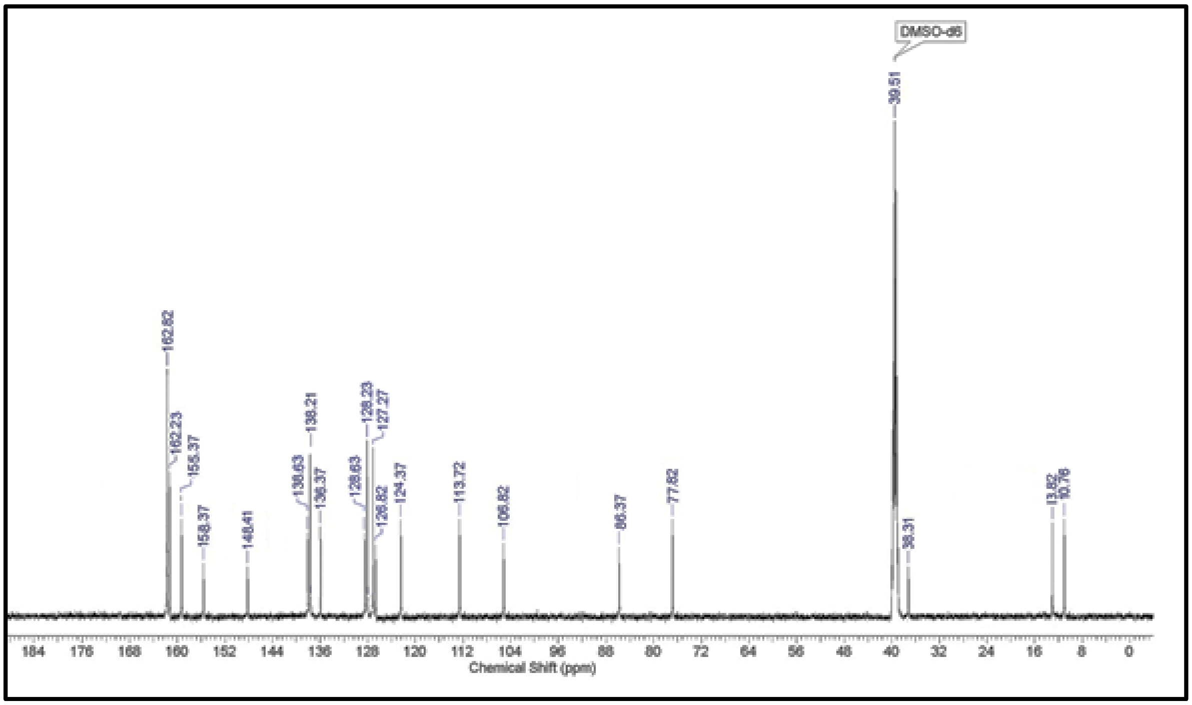

^{13}C NMR of compound 6

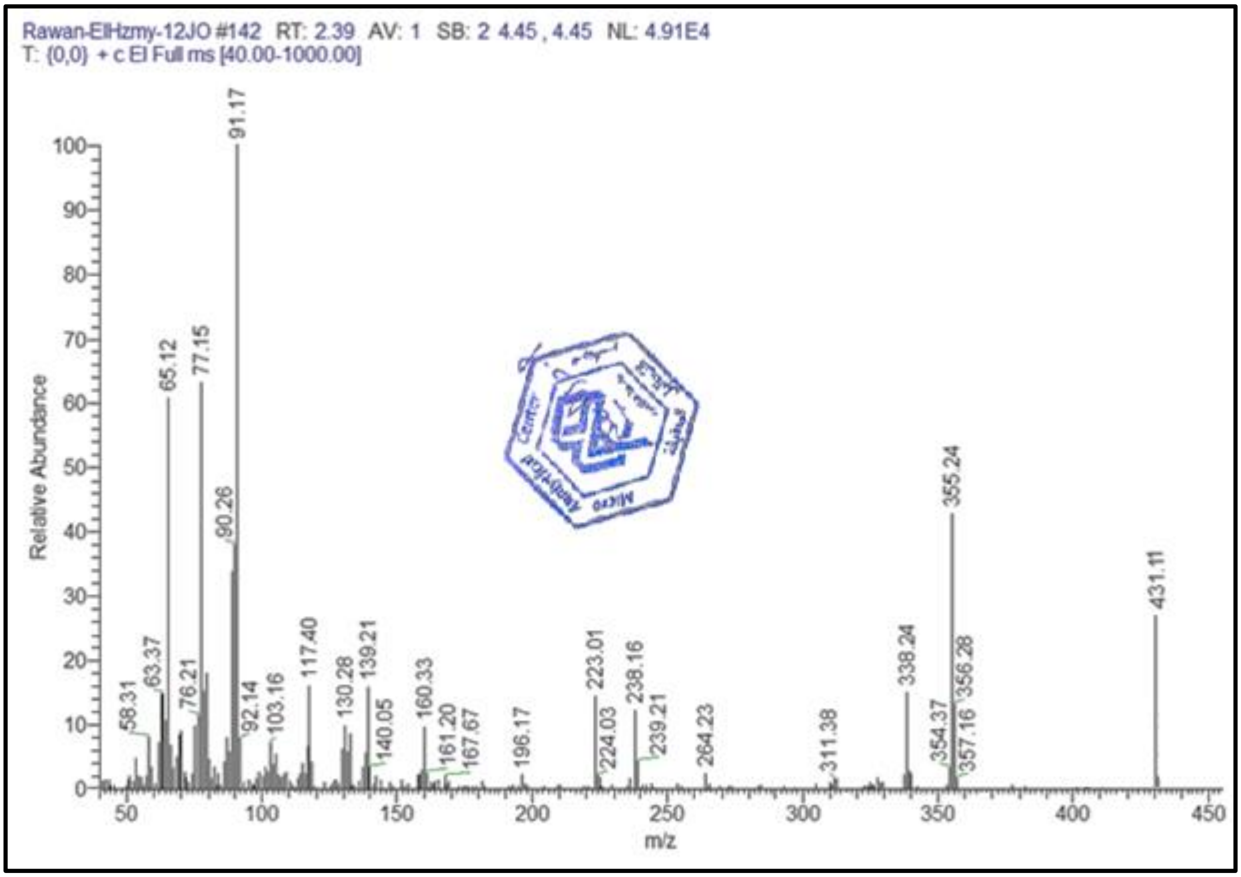

Mass of compound 9

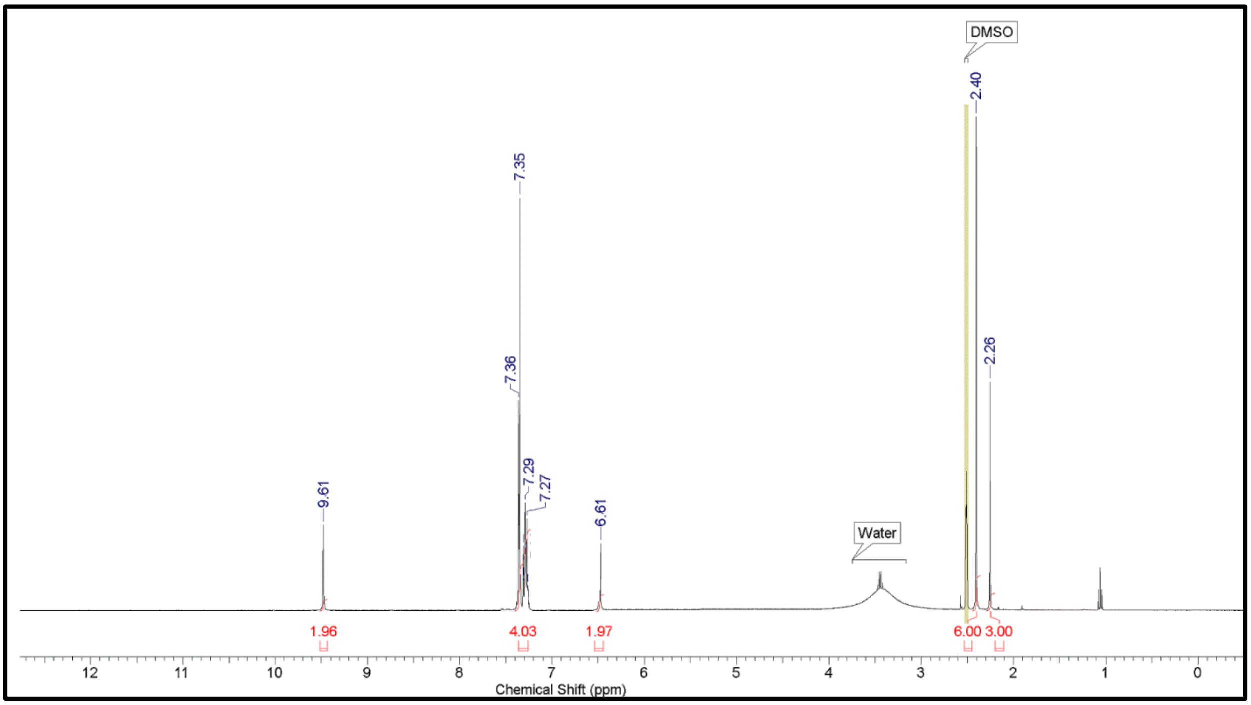

^{1}H NMR of compound 9

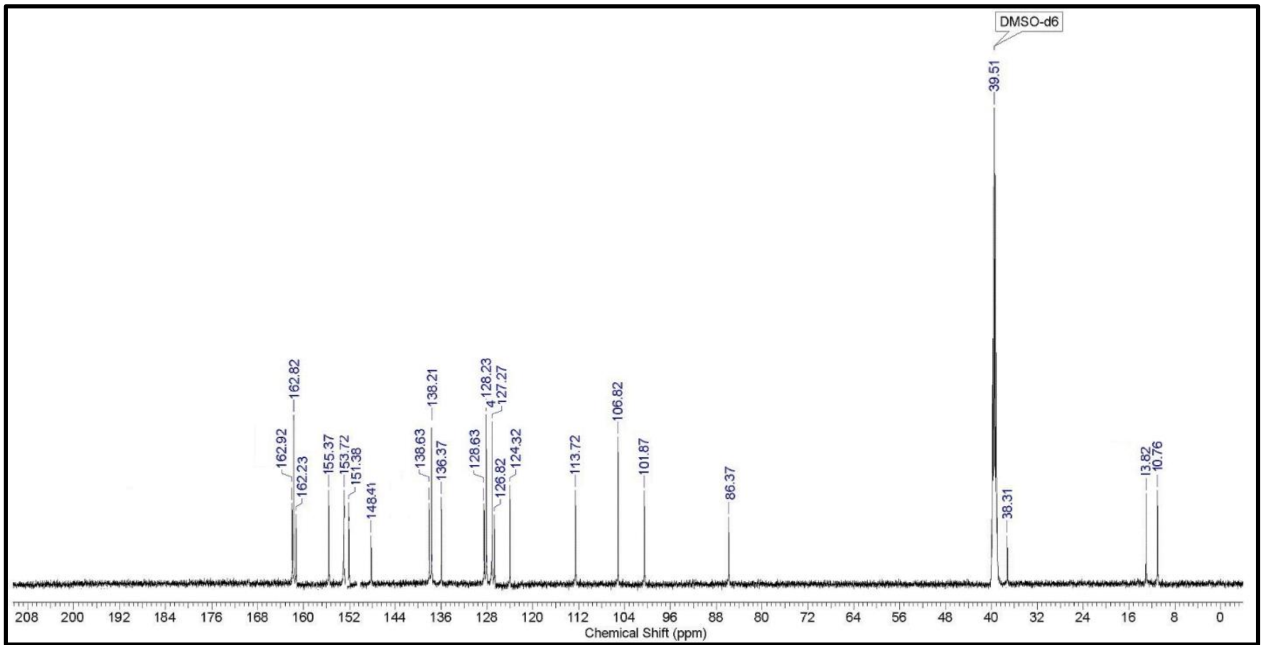

^{13}C NMR of compound 9

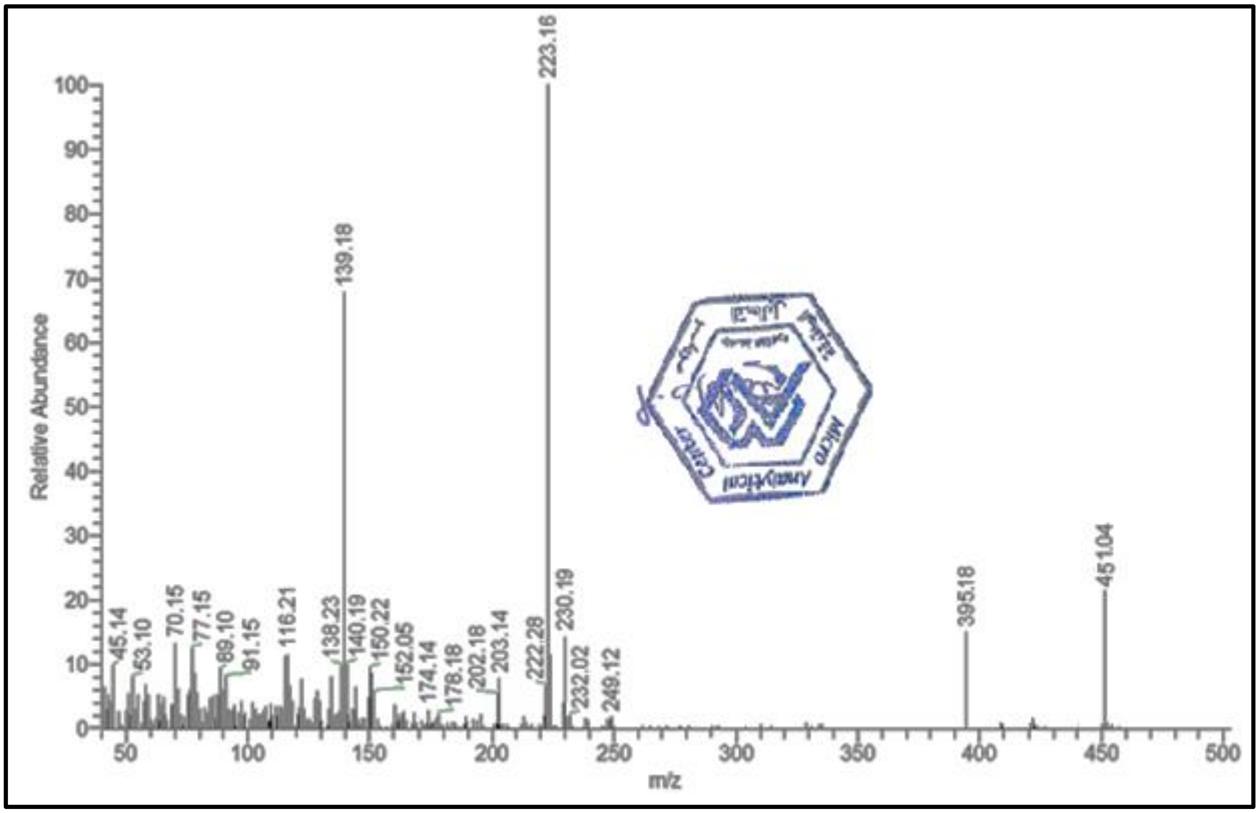

Mass of compound 13

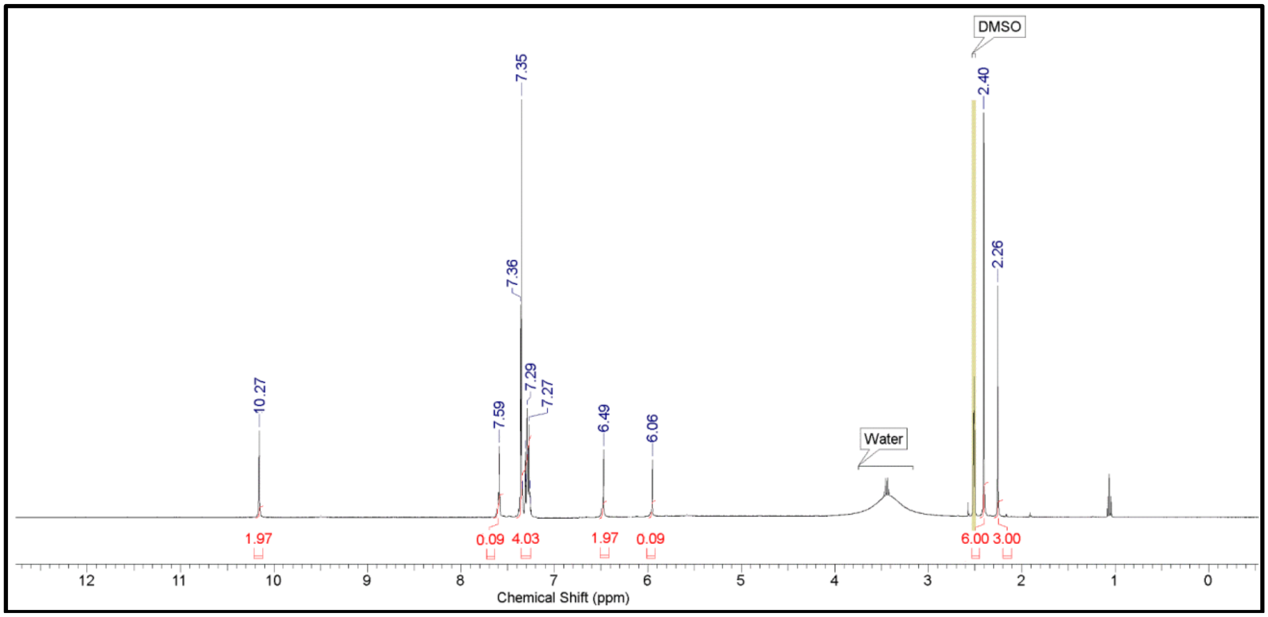

^{1}H NMR of compound 13

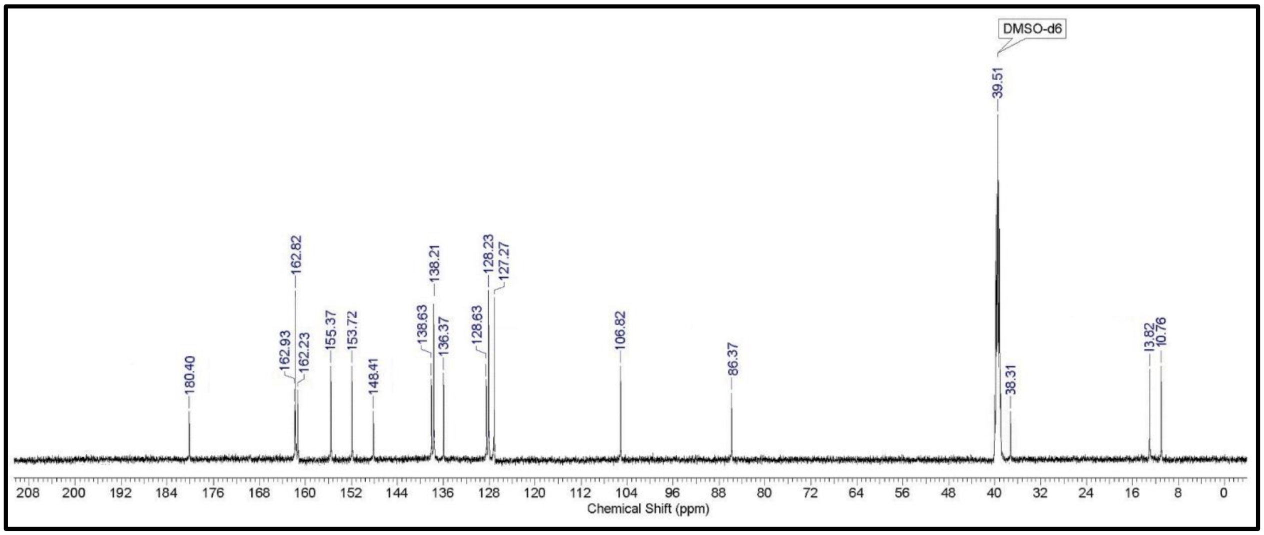

^{13}C NMR of compound 13

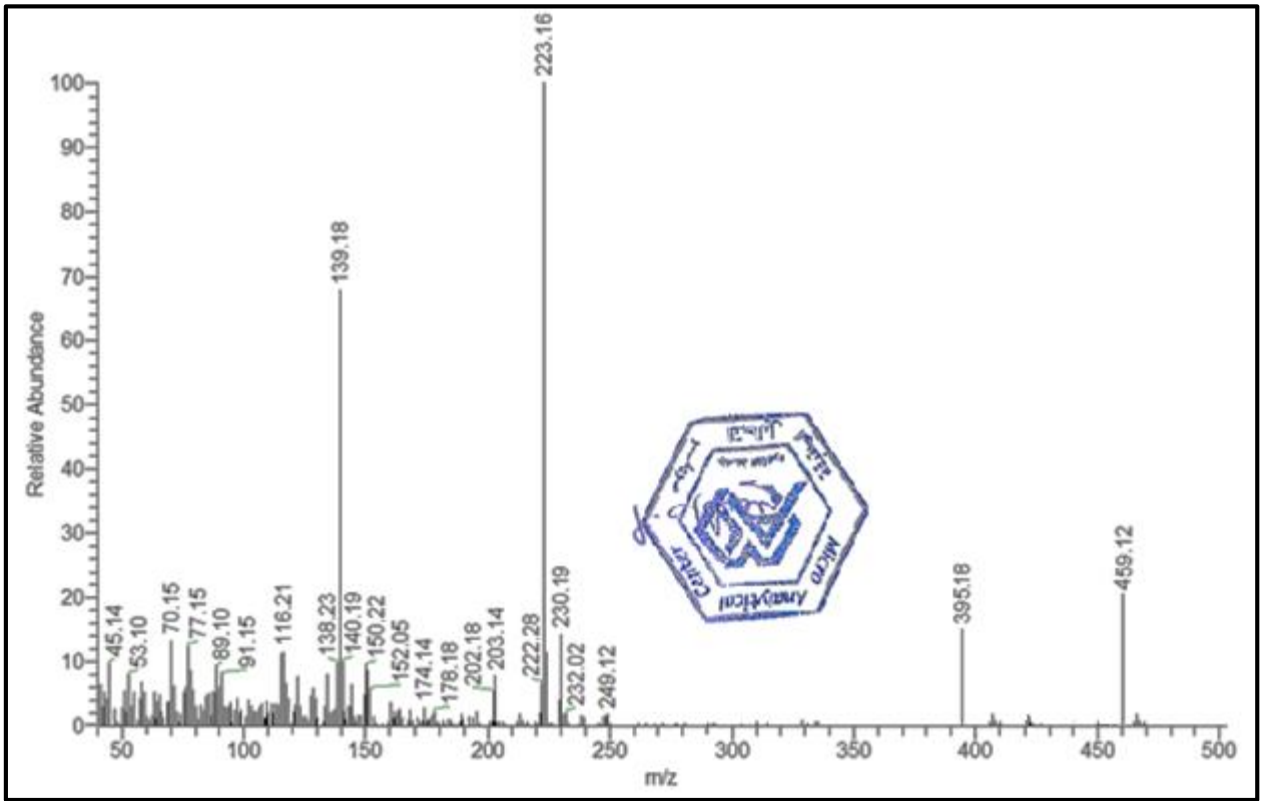

Mass of compound 16

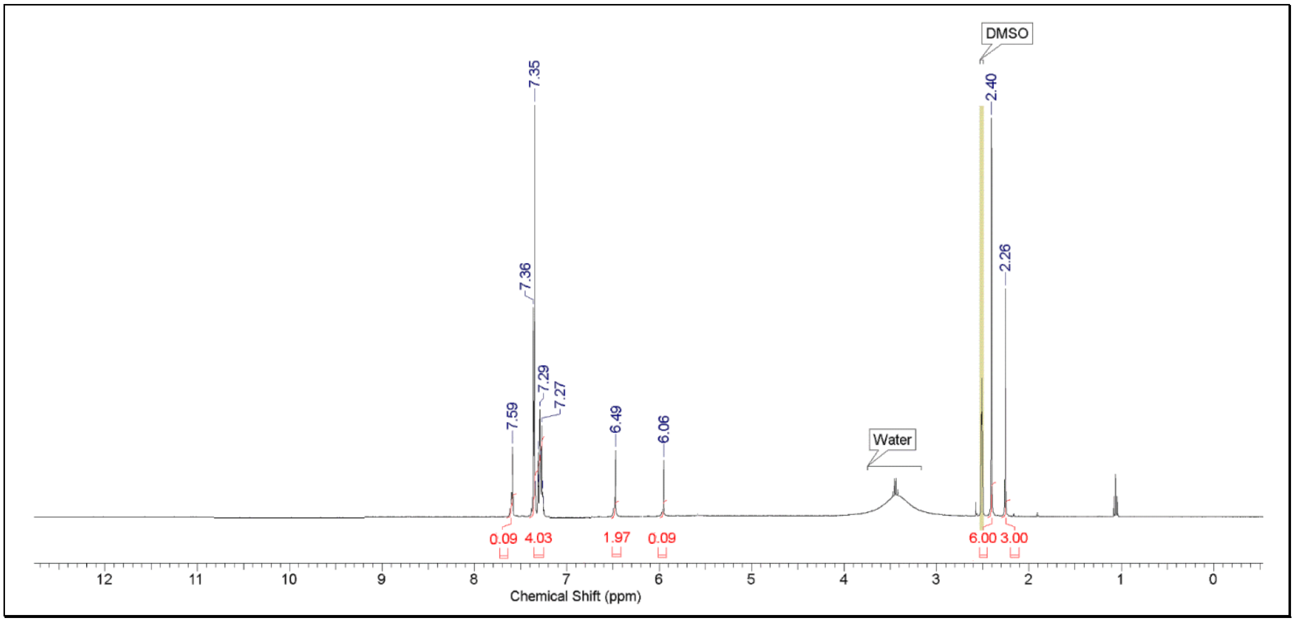

^{1}H NMR of compound 16

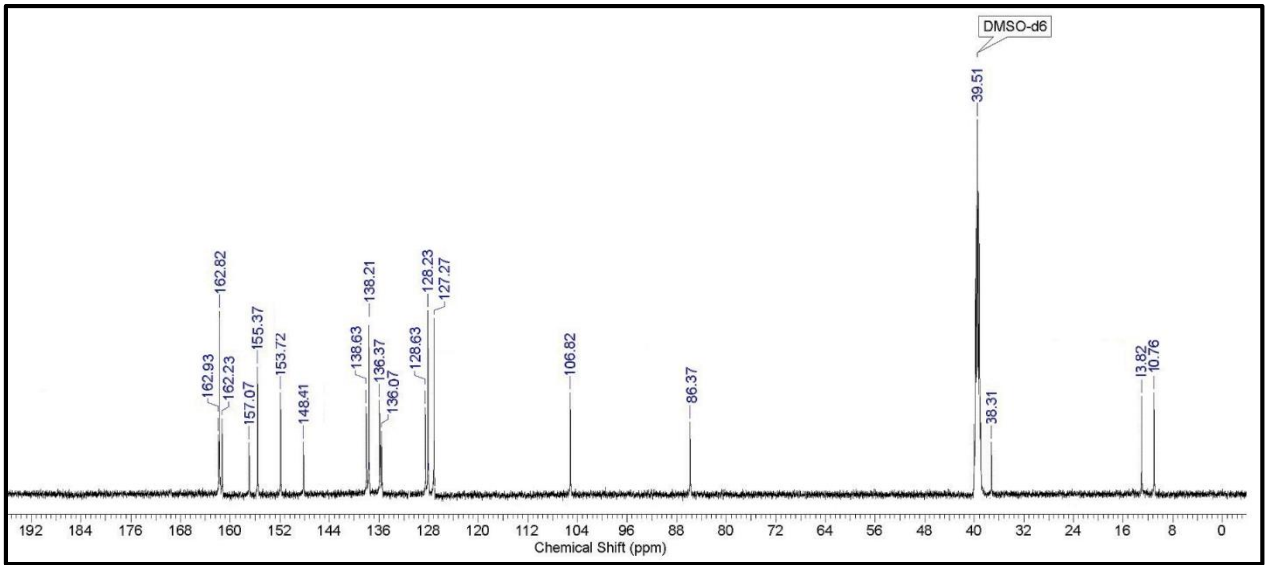

^{13}C NMR of compound 16
